# Systemic immune-inflammation index, SII, for prognosis of elderly patients with newly diagnosed tumors

**DOI:** 10.18632/oncotarget.24293

**Published:** 2018-10-19

**Authors:** Chan Li, Wei Tian, Feng Zhao, Meng Li, Qin Ye, Yuquan Wei, Tao Li, Ke Xie

**Affiliations:** ^1^ Department of Oncology, Southwest Medical University, Luzhou, Sichuan 646000, P.R. China; ^2^ Department of Radiation Oncology, Sichuan Cancer Hospital and Institute, Chengdu, Sichuan 610041, P.R. China; ^3^ Department of Operation Management, Sichuan Academy of Medical Sciences & Sichuan Provincial People's Hospital, Chengdu 610072, P.R. China; ^4^ Department of Biomedical Engineering, School of Medicine, University of Electronic Science and Technology of China, Chengdu, Sichuan 610054, P.R. China; ^5^ Department of Oncology, People's Hospital of Xinjin, Chengdu, Sichuan 611430, P.R. China; ^6^ Department of Oncology, Xinjin Precision Tumor Hospital, Chengdu, Sichuan 611430, P.R. China; ^7^ State Key Laboratory of Biotherapy and Cancer Center, West China Hospital, Sichuan University, Chengdu, Sichuan 610041, P.R. China; ^8^ School of Medicine, University of Electronic Science and Technology of China, Chengdu 611731, P.R. China

**Keywords:** systemic immune-inflammation index, predictive value, elderly patients, newly diagnosed, solid tumors

## Abstract

**Background:**

Cancer in the elderly has become a common problem due in part to the increase in life expectancy. Compared to younger counterparts, the biological characteristics of tumors and their responsiveness to therapy may differ in elderly patients, and the elderly also can have a decreased tolerance to anticancer therapy. In addition, there is less evidence from clinical trials to guide physicians in treating aged patients with solid tumors. Thus, we often face a dilemma as to how actively to treat these patients and it would be highly useful to have a simple and powerful indicator of their prognosis. In this paper we evaluated the predictive value of the Systemic Immune-inflammation Index, SII, in determining the one-year survival and tumor differentiation status in elderly patients with newly diagnosed solid tumors.

**Results:**

A high SII > 390×10^9^ cells/L was correlated with poor tumor differentiation (χ^2^ = 9.791, *P* = 0.002) and poor one-year survival (χ^2^ = 7.658, *P* = 0.006). Patients with low SII had improved survival and better tumor differentiation (Stage I-II). The SII was not associated with Ki-67 expression.

**Materials and Methods:**

Data from 119 patients, 70 to 89 years of age with newly diagnosed solid tumors in 2014 were retrospectively analyzed. The patients were divided into two groups according to age: (1) 70-75 years of age and (2) over 75 years of age. We calculated SII from the equation, SII = P x N/L, where P, N and L are the preoperative peripheral blood platelet, neutrophil and lymphocyte counts per liter respectively. The optimum cutoff point for SII for a favorable prognosis was determined to be 390×10^9^ cells/L. For evaluation of SII as a prognostic indicator, the patients were divided into high SII (> 390×10^9^ cells/L) and low SII (≤ 390×10^9^ cells/L) groups. Individual values were used to determine the relationship between SII and one-year survival, tumor differentiation and Ki-67 expression in the two age groups.

**Conclusions:**

SII was a robust indicator of tumor differentiation and one-year survival in elderly patients with newly diagnosed solid tumors. Patients in the high SII group showed poor tumor differentiation and poor prognosis compared to patients with a low SII score.

## INTRODUCTION

Cancer is a major international public health problem [1] and the incidence of cancer in the elderly has been increasing in part because of greater life expectancy. Studies have shown that by 2013 in the United States nearly 70% of all cancers will be diagnosed in adults 65 years or older [2]. Furthermore, compared to younger individuals, the physiological characteristics of cancers and their responsiveness to therapy are different in the elderly, and older patients can have a decreased tolerance for anticancer therapy [3]. Therefore, treating aged patients with solid tumors requires that doctors use a different set of predictors for therapeutic decisions based on NCCN (National Comprehensive Cancer Network) and ESMO Clinical Practice Guidelines. The data upon which these guidelines are based were drawn from classical clinical trials that are different from real world research. Since elderly tumor patients were seldom included in classical clinical trials it became apparent that therapeutic decisions based on such trials might not provide the necessary prognostic accuracy for effective treatment of aged patients with solid tumors. As physicians, we must balance the benefits of aggressive cancer treatment against the risks, and there is an urgent need to find a simple, accurate and robust prognostic indicator to evaluate the efficacy of a particular therapeutic regimen in elderly patients.

It is well known that cancer and inflammation are linked and the cellular immune system is key to the inflammatory response [4]. An increase in the neutrophil to lymphocyte ratio has been associated with an adverse prognosis in patients with cancer [5–11]. In addition, tumor cell survival and metastasis are both influenced by platelets [12–14]. A prognostic indicator based on counts of neutrophils, lymphocytes and platelets, then, is expected to be more robust than one based on only a single factor. In 2014, Hu et al. [15] developed an indicator that they called the Systemic Immune-inflammation Index, SII, to predict prognosis of patients after curative resection for hepatocellular carcinoma. The SII was calculated from preoperative counts of peripheral blood platelets (P), neutrophils (N) and lymphocytes (L) per liter according to the equation:

SII = P x N/L

The researchers tested the hypothesis that a high SII score (SII > 330×10^9^ cells/L) indicated poor outcome in these patients [15] and found it to be an accurate predictor of one-year survival and tumor differentiation. Other researchers questioned whether this SII cut-off value was appropriate for predicting prognosis in all cancer patients. Recent published evidence [16–19] suggests that SII is a useful and accurate independent prognostic indicator for all kinds of tumor patients.

Since 2014, we have been focusing on SII in aged patients with newly diagnosed solid tumors. To the best of our knowledge, no studies have reported the prognostic significance of the SII in elderly patients with newly diagnosed solid tumors. In this retrospective analysis, we evaluated the predictive value of SII for one-year survival and tumor differentiation in these patients.

## RESULTS

Patients' characteristics at baseline are shown in Table 1. The patients' ages ranged from 70 to 89 years and 49 (41.2%) were female and 70 (58.8%) were male. All patients had a body-mass index (BMI) of 18.5 kg/m^2^ or more. According to the Seventh Edition of the AJCC [20], 61 (51.26%) patients were in stages I-II and 58 (48.74%) patients were in stages III-IV. Comorbidities included diabetes mellitus, hypertension and coronary heart disease. We divided the patients into two groups according to age: 70 to 75 years of age and over 75 years of age. Patients in the two age groups were all comparable for gender, tumor number, comorbidity, ability to care for themselves (ECOG PS), tumor differentiation, one-year survival, blood albumin level and tumor resection. There were no significant differences of these parameters between the two age groups (all *P* > 0.05).

**Table 1:** Patients'(n=119) characteristics

Parameter	70≦ Age ≦75	Age >75	Total	*P*
	N (%)	N (%)	N (%)	
**Sex**				0.403
Male	36(55.4%)	34(63.0%)	70 (58.8%)	
Female	29(44.6%)	20(37.0%)	49 (41.2%)	
**Tumor number**				0.136
Single	63 (96.9%)	45(88.2%)	108 (93.1%)	
Multiple	2(3.1%)	6(11.8%)	8 (6.9%)	
**Comorbidity**				0.208
Yes	34(52.3%)	22(40.7%)	56(47.1%)	
No	31(47.7%)	32(59.3%)	63(52.9%)	
**ECOG PS**				0.243
0-1	63(56.2%)	49(43.8%)	112	
≥2	2(28.6%)	5(71.4%)	7	
**Tumor differentiation**				0.085
I-II	38(62.3%)	23(37.7%)	61	
III-IV	27(46.6%)	31(53.4%)	58	
**One-year survival**				0.105
Alive	55(57.9%)	40(42.1%)	95	
Dead	9(39.1%)	14(60.9%)	23	
**BMI (kg/m^2^)**				
<18.5	0	0	0	
≥18.5	64(55.2%)	52(44.8%)	116	
**Alb (g/l)**				0.076
<35.0	6(35.3%)	11(64.7%)	17	
≥35.0	59(58.4%)	42(41.6%)	101	
**Tumor resection**				0.077
Yes	63(57.3%)	47(42.7%)	110	
No	2(22.2%)	7(77.8%)	9	

Abbreviations: ECOG, Eastern Cooperative Oncology Group; PS, performance status; BMI, body mass index; Alb, albumin.

### Association of SII with tumor differentiation

Tumor differentiation (stage) was here defined according to the Seventh Edition of the AJCC [20]. As has been shown in many studies, the efficacy of anticancer treatment is directly related to tumor differentiation, so correct identification of tumor stage is critical for optimal therapeutic outcomes [23]. We examined whether there was a relationship between SII and tumor differentiation by using the Chi-square test or Fisher Exact test to compare the data from the two groups of elderly patients--high SII (> 390×10^9^ cells/L) and low SII (≤ 390×10^9^ cells/L). The better tumor differentiation (I-II) was 28 (71.79%) in patients in the low-SII group and 33 (41.25%) in patients in the high-SII group. In contrast, the poorest tumor differentiation (III-IV) was seen in only 11 (28.21%) of the low-SII patients while 47 (58.75%) of the high-SII patients were in stage III-IV. Table 2 shows that patients with low SII had a significantly better tumor differentiation than patients with high SII (*χ^2^* = 9.791, *P* = 0.002). Thus, our data show that SII provides a robust indicator of tumor differentiation potentially useful in creating a therapeutic regimen.

**Table 2:** SII and tumor differentiation

Parameter	SII(×10^9^ cells/L)		Total	*χ^2^*	*P*
	≤390 (Low)	>390 (High)			
**Tumor differentiation**				9.791	0.002
I-II	28(71.79%)	33(41.25)	61		
III-IV	11(28.21%)	47(58.75%)	58		
**Total**	39	80	119		

### Correlation between SII and one-year survival

The correlation between the pre-therapeutic SII and one-year survival is shown in Table 3. One-year survival was calculated from the date at which the tumor was diagnosed. Elderly patients (70 to 75 yoa and >75 yoa) with newly diagnosed solid tumors (n = 118) were divided into two groups using the SII cut-off of 390 × 10^9^ cells/L). There were 39 (33.05%) patients with low SII and 79 (66.95%) patients with high SII. The difference between the two groups was analyzed by Chi-square and Fisher Exact test (*χ^2^* = 7.658, *P* = 0.006). The one-year survival rate was 94.87% in low-SII patients and 73.42% in high-SII patients, the high-risk group. These results further demonstrate that SII is a sensitive and useful clinical parameter to predict the one-year survival of patients 70 yoa or older with newly diagnosed solid tumors.

**Table 3:** SII and one-year survival

Parameter	SII(×10^9^ cells/L)		Total	*χ^2^*	*P*
	≤390 (Low)	>390 (High)			
**One-year survival**				7.658	0.006
Alive	37(94.87%)	58(73.42%)	95		
Dead	2(5.13%)	21(26.58%)	23		
**Total**	39	79	118		

### Correlation between SII and Ki-67 expression

As a cell proliferation marker, Ki-67 has prognostic value in human malignancies independent of other parameters [22]. The relationship between the pre-therapeutic SII and Ki-67 was investigated, and the data shown in Table 4. The *t* test was used to compare the means of the two groups. SII was found to be not associated with Ki-67 (*t* = 0.282, *P* = 0.778).

**Table 4:** Ki-67 and SII

Parameter	SII (×10^9^ cells/L)	N	Mean	SD	*t*	*P*
					0.282	0.778
**Ki-67**	≦390 (Low)	39	0.472	0.274		
	>390 (High)	80	0.459	0.253		

### Correlation between SII and age

We wanted to know whether or not the >75 year-old patients showed an age-related difference in SII. The Chi-square test was used to compare the means of the two groups, and there was no significant difference in SII results for the two age groups (Table 5, χ^2^ = 0.443, P = 0.505).

**Table 5:** SII and age

1Parameter	70≦ Age ≦75	Age >75	Total	*χ^2^*	*P*
**SII(×10^9^ cells/L)**				0.443	0.505
**≤390(Low)**	23(35.38%)	16(29.63%)	39		
**>390(High)**	42(64.62%)	38(70.37%)	80		
**Total**	65	54	119		

## DISCUSSION

The goal of an anticancer therapeutic regimen is to treat the cancer as aggressively as necessary to eliminate it without undue risk to the patient. This is difficult with the elderly cancer patient because the clinical studies physicians use as guides often do not include them. One critical piece of information that would aid in creating an optimal treatment program would be the individual patient's prognosis. In this paper, we explored for the first time the value of the SII in predicting one-year survival and tumor differentiation in elderly patients with newly diagnosed solid tumors. A prognostic indicator called the systemic immune-inflammation index (SII) was developed in 2014 [15] and is calculated from peripheral blood counts of platelets, neutrophils and lymphocytes prior to treatment using the equation:

SII = P x N/L where P, N and L are the cell counts per liter of peripheral blood for platelets, neutrophils and lymphocytes.

It was found that cancer patients with SII >330 × 10^9^ cells/L had a poorer prognosis than those with a lower SII score [15]. The physiological processes underlying the SII are not fully understood but may involve a number of factors. Neutrophils are associated with angiogenesis-regulating chemokines, growth factors and proteases and this could affect the tumor's blood supply and growth rate [24–27]. One of the main factors in a poor prognosis is metastasis of tumor cells. Circulating tumor cells, CTCs, may associate with platelets as they travel through the blood and this interaction can protect the CTCs from hemodynamic shear forces and NK cell killing [28, 29, 30]. Platelets can also secrete chemokines and cytokines that promote proliferation of metastasized tumor cells [31]. Circulating lymphocytes can inhibit proliferation and metastasis and an elevated preoperative lymphocyte count was recognized as a better outcome in resected pancreatic ductal adenocarcinoma [32, 33].

Additional research has confirmed that the SII can serve as an important prognostic marker in patients with other types of tumor such as small-cell lung cancer, renal cell cancer, metastatic castration-resistant prostate cancer, squamous cell carcinoma of the esophagus [16–19]. This means that the SII can be used as a prognostic indicator in ‘basket’ trials in which several different types of cancer all having the same gene mutation are studied [34]. Thus we can use SII with a certain targeted therapy to treat a variety of tumors.

Balancing the costs and benefits of a tumor therapy program is important and the fact that SII can be easily determined in a clinic or testing lab from routine blood work makes it a simple and inexpensive addition to the patient work-up. However, several limitations in this study need to be addressed before widespread use of SII can be accepted. The data were drawn retrospectively from a relatively small sample. We plan to repeat the study prospectively using a much larger population and expanding the age range. This will provide a more accurate and statistically verifiable data pool to test the clinical predictive value of SII.

## PATIENTS AND METHODS

### Selection/exclusion criteria

Data from a group of 119 patients, aged 70 to 89 years, with newly diagnosed solid tumors in 2014 were used in this retrospective analysis. The following were set as the inclusion criteria of the study: age ≥ 70 years and newly diagnosed solid tumor by pathology without history of cancer. Exclusion criteria of this study were: age < 70 years, history of cancer, recurrent tumors, or hematologic disease.

### Systemic immune-inflammation index

The SII was defined as follows: SII = *P* × *N*/*L*, where *P*, *N*, and *L* were the pre-therapeutic peripheral blood platelet, neutrophil, and lymphocyte counts in cells/L in the elderly patients with newly diagnosed solid tumors, respectively [15]. The SPSS software was used for analysis of the data to determine the optimal cutoff value of SII, which was found to be 390×10^9^ cells/L. Consequently, the patients were divided into high SII (> 390×10^9^ cells/L) and low SII (≤ 390×10^9^ cells/L) groups for evaluating the prognostic usefulness of SII.

### Clinical parameters

We compared high and low SII groups in term of one-year survival, tumor differentiation, and Ki-67 expression. Tumor differentiation (stage) was defined according to the Seventh Edition of the AJCC [20]. The one-year survival was assessed by the medical records and we also noted the patients' living conditions through the ID card number. The Ki-67 protein is a cellular marker for proliferation [21] that is present during the cell cycle in G_1_, S, G_2_, and mitosis but is absent from resting cells in G_0_ [21]. Ki-67 has independent prognostic value in human malignancies [22]. We measured Ki-67 levels by immunohistochemistry [monoclonal antibody kit and ZSGB-BIO, Origene] on pathology specimens. All these data were used to determine the relationship between SII and tumor differentiation, the one-year survival and Ki-67 expression.

### Statistical analysis

We divided the 119 aged patients with newly diagnosed solid tumors into two groups according to age: 70-75 years of age and over 75 years of age. The Chi-square test or Fisher Exact test was used to compare the data from the two groups and determine whether differences were statistically significant. The SPSS 17.0 statistical software was used for analysis of the data to determine the optimal cutoff value of SII for a favorable prognosis, which was found to be 390×10^9^ cells/L. Using this cutoff value, we divided the patients into two SII groups: low, SII ≤ 390 ×10^9^ cells/L or high, SII > 390 ×10^9^ cells/L for subsequent analysis. To determine the relationship between SII and one-year survival or tumor differentiation, the data from the two groups were analyzed statistically by Chi-square or Fisher Exact test. Association between Ki-67 expression and SII was determined using the *t* test and α<0.05 was taken as evidence of a statistically significant difference.

## CONCLUSIONS

Our data verified that SII could be used as an independent prognostic factor and significantly correlate with tumor differentiation and one-year survival in a population of newly diagnosed elderly cancer patients. Thus SII is proposed as a convenient and low-cost blood-derived prognostic test in aged patients with newly diagnosed solid tumors.
